# Testosterone treatment and the risk of aggressive prostate cancer in men with low testosterone levels

**DOI:** 10.1371/journal.pone.0199194

**Published:** 2018-06-22

**Authors:** Thomas J. Walsh, Molly M. Shores, Chloe A. Krakauer, Christopher W. Forsberg, Alexandra E. Fox, Kathryn P. Moore, Anna Korpak, Susan R. Heckbert, Steven B. Zeliadt, Chloe E. Kinsey, Mary Lou Thompson, Nicholas L. Smith, Alvin M. Matsumoto

**Affiliations:** 1 University of Washington, Seattle, Washington, United States of America; 2 VA Puget Sound Health Care System, Seattle, Washington, United States of America; 3 Group Health Research Institute, Group Health Cooperative, Seattle, Washington, United States of America; 4 Division of Gerontology & Geriatric Medicine, Department of Medicine, University of Washington School of Medicine, Seattle, Washington, United States of America; 5 Geriatric Research, Education and Clinical Center, VA Puget Sound Health Care System, Seattle, Washington, United States of America; University of South Alabama Mitchell Cancer Institute, UNITED STATES

## Abstract

**Purpose:**

Testosterone treatment of men with low testosterone is common and, although relatively short-term, has raised concern regarding an increased risk of prostate cancer (CaP). We investigated the association between modest-duration testosterone treatment and incident aggressive CaP.

**Materials and methods:**

Retrospective inception cohort study of male Veterans aged 40 to 89 years with a laboratory-defined low testosterone measurement from 2002 to 2011 and recent prostate specific antigen (PSA) testing; excluding those with recent testosterone treatment, prostate or breast cancer, high PSA or prior prostate biopsy. Histologically-confirmed incident aggressive prostate cancer or any prostate cancer were the primary and secondary outcomes, respectively.

**Results:**

Of the 147,593 men included, 58,617 were treated with testosterone. 313 aggressive CaPs were diagnosed, 190 among untreated men (incidence rate (IR) 0.57 per 1000 person years, 95% CI 0.49–0.65) and 123 among treated men (IR 0.58 per 1000 person years; 95% CI 0.48–0.69). After adjusting for age, race, hospitalization during year prior to cohort entry, geography, BMI, medical comorbidities, repeated testosterone and PSA testing, testosterone treatment was not associated with incident aggressive CaP (HR 0.89; 95% CI 0.70–1.13) or any CaP (HR 0.90; 95% CI 0.81–1.01). No association between cumulative testosterone dose or formulation and CaP was observed.

**Conclusions:**

Among men with low testosterone levels and normal PSA, testosterone treatment was not associated with an increased risk of aggressive or any CaP. The clinical risks and benefits of testosterone treatment can only be fully addressed by large, longer-term randomized controlled trials.

## Introduction

Serum testosterone declines with age, such that 20–40% of men over 40 years old have low testosterone levels[1, 2] with 2–6% having symptoms of androgen deficiency and clinical hypogonadism.[3–5] Despite clinical guidelines recommending testosterone treatment only for men with symptomatic androgen deficiency and consistently low testosterone levels,[6] testosterone therapy has been used increasingly to treat men with a single low testosterone level.[7, 8] When initiated, testosterone treatment is often short-term, usually for an average of 16 months or less[9–12]. Little is known about the safety of testosterone use in this clinical context. An increase in prostate specific antigen (PSA) within the first 12 months of initiating testosterone treatment[13, 14], combined with the association between testosterone treatment and increases in prostate size, raises concerns about the possible increase in prostate cancer risk.[15–18] Testosterone treatment could increase the near-term risk of prostate cancer by stimulating an occult tumor or it may impact risk over a longer period of time. Randomized trials that have been conducted to date have been underpowered to detect risk for prostate cancer.[19–22] Meta-analyses of these testosterone treatment trials have not found increased risk of prostate cancer but have identified an increased risk for prostate biopsy.

We used data from the Veterans Health Administration (VHA) to determine if current patterns of testosterone use in Veterans are associated with adverse prostate cancer outcomes. We hypothesized that modest-duration testosterone treatment relative to no treatment is associated with an increase in prostate cancer risk and that, among testosterone users, a larger cumulative dose is associated with a higher risk.

## Methods

### Study design

We created an inception cohort study of men with low serum testosterone followed for initiation of testosterone treatment and incident aggressive prostate cancer. We utilized data from the United States Department of Veterans Affairs VHA, a large, electronically-integrated health care organization that provides medical care to eligible Veterans. The VHA Institutional Review Board approved this study.

### Study cohort

Men aged 40–89 years with a laboratory-defined, low testosterone measurement who received outpatient care in any VHA facility between January 1, 2002 and December 31, 2011, had at least 2 in-person VA clinic visits in the year prior to cohort entry, and PSA testing within 6 months of the low testosterone level (and prior to testosterone treatment) were eligible to enter the cohort (Appendix A). Cohort entry was the date of the first low testosterone level or the date of the qualifying PSA test, whichever occurred later. We excluded men with any history of testosterone treatment in the year prior to cohort entry, prevalent or past prostate or breast cancer, PSA ≥ 4.0 ng/dL, or a history of prostate biopsy.

We required that men survive one year following cohort entry because we assumed that there is increased potential for diagnosis of pre-existing prostate cancer in the first year[23] and that, if present, the effect of testosterone on prostate cancer progression exceeds one year. Men were followed until prostate cancer diagnosis, or censoring due to death, loss to follow-up (730 days after their last VA clinic visit), or end of study (September 30, 2012).

### Data sources

We collected data from two sources: the Corporate Data Warehouse (CDW), and the Veterans Affairs Central Cancer Registry (VACCR) (URL: https://www.hsrd.research.va.gov/for_researchers/vinci/default.cfm; email: VINCI@VA.GOV).

The records obtained from these databases provided demographics, laboratory results and dates, prescription data, dates of clinic visit and hospitalizations, *International Classification of Diseases* ninth edition (ICD-9) diagnostic and procedure codes, and *Current Procedural Terminology* (CPT) procedure codes (Appendices A, B, and C).

### Serum testosterone measurements

Serum testosterone tests were identified using the Logical Observation Identifiers Names and Codes (LOINC, see Appendix A). Men were classified as having low serum testosterone if they had a total testosterone, free testosterone, free/total testosterone, free testosterone index, or bioavailable testosterone test flagged as low in the reference range used by the specific testing laboratory and used by ordering providers in day-to-day clinical decision making.

### Testosterone exposure

Testosterone prescriptions were identified using VA National Drug Internal Entry Numbers (IENs).

Data included the testosterone formulation (intramuscular injection, patch, or gel), initial prescription date, refill dates, dose, and amount dispensed (Appendices A and B). Treatment initiation was defined as the first prescription for testosterone following cohort entry. We assessed testosterone levels during treatment by averaging all levels measured after the initiation of treatment in testosterone-treated and after cohort entry in untreated men.

Two methods were used to model the association between time-varying testosterone exposure and prostate cancer risk: 1) Binary exposure where men were regarded as not treated until they received their first testosterone prescription and as treated thereafter, even if treatment was intermittent or later discontinued (analogous to an intention-to-treat analysis); and 2) Time-varying cumulative dose, calculated by summing the amount in mg of testosterone *delivered* (e.g., 5 gm dose of testosterone gel = 50 mg of testosterone applied = 5 mg of testosterone delivered) that was dispensed in all filled prescriptions.

### Outcomes

The primary and secondary outcomes were the first occurrence of *aggressive* or *any* prostate cancer, respectively, between January 1, 2002 and September 30, 2012. All prostate cancers were histologically confirmed in the VACCR which provided cancer histology, Gleason score, clinical stage, and PSA at the time of diagnosis (Appendix C).

Aggressive cancer was defined by the presence one of the following: Surveillance Epidemiology and End Results (SEER) Summary Stage (distant metastases) = 7, American Joint Committee on Cancer Stage Group = 4, any metastasis, tumor grade = 4, Gleason score ≥ 8, or PSA at diagnosis ≥ 20 ng/dL.

### Medical comorbidities

Medical comorbidities were determined using ICD-9 diagnosis and procedure codes and CPT procedure codes, and medications and lab results that indicated specific conditions. A total of 26 medical conditions and hospitalization in the 365 days prior to cohort entry were determined *a priori* to characterize medical comorbidity (Appendix B, Table 1).

**Table 1:** Baseline characteristics of men with low testosterone, 2002 to 2011.

	Treated	Not Treated	All
	(N = 58,617)	(N = 88,976)	(N = 147,593)
**Age at Baseline (years)**			
40–44	2,274 (4%)	2,461 (3%)	4,735 (3%)
45–49	4,353 (7%)	5,292 (6%)	9,645 (7%)
50–54	8,346 (14%)	10,869 (12%)	19,215 (13%)
55–59	13,720 (23%)	18,485 (21%)	32,205 (22%)
60–64	15,116 (26%)	22,585 (25%)	37,701 (26%)
65–69	7,305 (12%)	12,193 (14%)	19,498 (13%)
70–74	3,785 (6%)	7,813 (9%)	11,598 (8%)
75–79	2,330 (4%)	5,488 (6%)	7,818 (5%)
80–85	1,077 (2%)	2,778 (3%)	3,855 (3%)
≥ 85	311 (1%)	1,012 (1%)	1,323 (1%)
**Region of U.S.**			
Western	14,730 (25%)	20,776 (23%)	35,506 (24%)
Upper Midwest	5,437 (9%)	9,288 (10%)	14,725 (10%)
Upper Middle and Eastern	9,493 (16%)	15,870 (18%)	25,363 (17%)
Northeastern	6,280 (11%)	10,928 (12%)	17,208 (12%)
Southern	22,677 (39%)	32,114 (36%)	54,791 (37%)
**BMI (kg/m**^**2**^**)**			
0–18.4	361 (1%)	809 (1%)	1,170 (1%)
18.5–24.9	4,867 (8%)	10,607 (12%)	15,474 (10%)
25–29.9	15,995 (27%)	27,215 (31%)	43,210 (29%)
30–34.9	18,820 (32%)	26,977 (30%)	45,797 (31%)
≥ 35	18,574 (32%)	23,368 (26%)	41,942 (28%)
**Race**			
White	44,738 (76%)	64,487 (72%)	109,225 (74%)
Black	7,529 (13%)	13,649 (15%)	21,178 (14%)
Other	6,350 (11%)	10,840 (12%)	17,190 (12%)
**Baseline Comorbidities**			
BPH	18,118 (31%)	28,558 (32%)	46,676 (32%)
Cardiac Arrhythmia	11,441 (20%)	19,042 (21%)	30,483 (21%)
Cardiomyopathy	1,839 (3%)	3,438 (4%)	5,277 (4%)
CAD^a^ (hard outcomes^b^)	7,333 (13%)	12,578 (14%)	19,911 (13%)
CAD (soft outcomes^b^)	17,732 (30%)	29,142 (33%)	46,874 (32%)
Chronic Heart Failure	5,109 (9%)	9,343 (11%)	14,452 (10%)
Chronic Liver Failure	2,179 (4%)	3,837 (4%)	6,016 (4%)
Chronic Lung Disease	14,582 (25%)	22,516 (25%)	37,098 (25%)
Chronic Pain	2,755 (5%)	3,483 (4%)	6,238 (4%)
CVD^c^ (hard outcomes^b^)	859 (1%)	1,786 (2%)	2,645 (2%)
CVD (soft outcomes^b^)	4,003 (7%)	7,446 (8%)	11,449 (8%)
Diabetes	25,777 (44%)	39,644 (45%)	65,421 (44%)
Sexual dysfunction^d^	35,078 (60%)	42,831 (48%)	77,909 (53%)
Frailty	389 (1%)	745 (1%)	1,134 (1%)
Hyperlipidemia	21,156 (36%)	30,630 (34%)	51,786 (35%)
Hypertension	46,632 (80%)	70,704 (79%)	117,336 (80%)
Major Depression	12,829 (22%)	16,046 (18%)	28,875 (20%)
Malignancy	2,385 (4%)	4,405 (5%)	6,790 (5%)
Morbid Obesity	6,583 (11%)	8,220 (9%)	14,803 (10%)
Osteoporosis	3,004 (5%)	4,839 (5%)	7,843 (5%)
PVD^e^	5,885 (10%)	10,442 (12%)	16,327 (11%)
Polycythemia	395 (1%)	452 (1%)	847 (1%)
Sleep Apnea	14,359 (25%)	17,311 (19%)	31,670 (21%)
Smoking	20,074 (34%)	31,593 (36%)	51,667 (35%)
TBI^f^	1,607 (3%)	2,360 (3%)	3,967 (3%)
DVT^g^/PE^h^	2,194 (4%)	3,603 (4%)	5,797 (4%)
**Total Number of Medical Comorbidities**			
0 to 3	19,792 (33.76%)	31,984 (35.95%)	51,776 (35.08%)
4 to 7	29,538 (50.39%)	42,797 (48.10%)	72,335 (49.01%)
8 to 11	8,158 (13.92%)	12,146 (13.65%)	20,304 (13.76%)
12 to 15	1,080 (1.84%)	1,931 (2.17%)	3,011 (2.04%)
16 to 19	49 (0.08%)	117 (0.13%)	166 (0.11%)
At least 20	0 (0.00%)	1 (0.00%)	1 (0.00%)

^a^ Coronary Artery Disease

^b^ See Appendix B for definitions of hard and soft outcomes for CAD and CVD

^c^ Cerebrovascular Disease

^d^ Sexual Dysfunction, including erectile dysfunction, reduced libido, and hypoactive sexual desire disorder

^e^ Peripheral Vascular Disease

^f^ Traumatic Brain Injury

^g^ Deep Vein Thrombosis

^h^ Pulmonary Embolism

### Statistical analysis

We performed Cox proportional hazards regression to estimate the adjusted associations of testosterone treatment with aggressive and any prostate cancer. Time-varying testosterone exposure was modeled: 1) as a dichotomous variable (treated/not treated) with follow-up starting one year after cohort entry; and 2) by cumulative testosterone dose during follow up in 5 categories among new testosterone users (1-399mg, 400-799mg, 800-1599mg, 1600-3199mg, and ≥ 3200mg), with follow-up starting one year after their first testosterone prescription. We performed baseline adjustment for age, race (White, Black, other), site of care, BMI, hospitalization in year prior to cohort entry, and 26 medical comorbidities (Table 1, Appendix B) and time-varying adjustment for medical comorbidities, repeated testosterone testing and PSA screening intensity.

We defined the “repeat testosterone level” variable as: repeated testosterone level low, repeated testosterone level not low or no repeat testosterone level. This measure was included because guidelines recommend at least two low testosterone levels prior to starting testosterone treatment.[6]

Given the importance of PSA screening on the detection of prostate cancer, we assessed PSA testing across all years of study and we defined a time-varying “PSA screening intensity” variable as:: PSA screened in the last 6 months, screened in the last 6–12 months, or not screened in the last 12 months.

In analyses for aggressive and any prostate cancer, we adjusted for the number of comorbidities and presence/absence of individual comorbidities, respectively. In cumulative dose analyses, restricted to treated men, we adjusted for quintiles of the time from entry into the analytic cohort to treatment initiation to account for postponement of treatment.

Given that older men may be less likely to be screened for prostate cancer, we performed sensitivity analyses, where we separately considered men aged under 70 years at cohort entry and men aged 70 years or older at cohort entry. In secondary analyses, we considered the effect of testosterone exposure separately for intramuscular and topical treatment. Because prostate cancer initiation, progression, and clinical recognition are slow, we assessed the association of testosterone exposure with prostate cancer for men with a longer duration of follow-up by conducting two additional analyses: commencing follow-up at 3 and at 5 years after cohort entry.

Analyses were carried out using Stata Version 15.0. StataCorp. 2015. *Stata Statistical Software*: *Release 15*. College Station, TX: StataCorp LP.

## Results

We identified 301,003 men aged 40 to 89 with low testosterone flagged by the testing laboratory and with complete lab data. After *a priori* exclusions were applied, 147,593 (49%) men remained in the low-testosterone analysis cohort. During follow-up, 56,833 men initiated testosterone treatment (S1 Fig).

Most low testosterone measurements were total testosterone (80.9%) and free testosterone (14.5%). The median follow-up time for all men was 3.0 years (range, 1 day to 9.8 years) with 25% of men having follow up for > 5.9 years. Total follow-up time was slightly longer for treated men vs. untreated men (median, 3.2 vs. 2.8 years, respectively).

We identified 1,352,977 prescriptions for testosterone, of which 862,344 (63.7%) were intramuscular (IM), 361,644 (26.7%) were topical patches, and 128,989 (9.5%) were topical gels. Of the 56,833 men treated with testosterone, 22,738 (40.0%) were treated solely with IM testosterone, 21,746 (38.3%) solely with topical testosterone, and 12,349 (21.7%) with both. The mean cumulative duration of testosterone treatment was 27.0 (SD 27.3) months. The median cumulative duration of testosterone treatment was 17.3 (interquartile range (IQR) 6.6 to 36.8) months. The median number of PSA tests per year in testosterone treated and untreated men were 0.9 (IQR 0.4, 1.4) and 0.7 (IQR 0.0, 1.1) respectively.

There was a high burden of medical comorbidities at baseline among cohort members (Table 1). There was little difference in the characteristics of men treated and not treated with testosterone, except sexual dysfunction, which was more prevalent among treated than untreated men (60% vs. 48%, respectively).

Repeat testosterone testing was more common among testosterone-treated men, with testosterone testing in 15,794 (69.5%) and 14,385 (66.2%) in IM- and topical-treated, respectively, versus 31,540 (35.5%) in untreated men. Untreated men had higher baseline testosterone levels than testosterone-treated men. At follow-up testosterone measurement, testosterone levels were higher among all men, including those who were untreated, and there was little difference in follow-up mean serum testosterone level between untreated and topical testosterone-treated men. However, IM testosterone-treated men had a mean serum testosterone level that was approximately 2-fold greater than that of untreated and topical testosterone-treated men (Table 2).

**Table 2:** Mean total serum T levels at cohort entry and follow-up among men treated and not treated with T.

	Total Testosterone Level (ng/dL)		
	No T Treatment	Topical T Treatment	Intramuscular T Treatment
N	31,540	14,385	15,794
Baseline T, Mean (SD)	217.2 (128.1)	179.4 (81.4)	178.5 (78.3)
Follow-up T, Mean (SD)^a^	280.3 (158.6)	272.30 (154.4)	365.8 (310.1)
Change in T, Mean (95% CI)	63.1 (61.4–64.7)	92.9 (90.5–95.4)	187.3 (182.4–192.1)

^a^ Among treated men, T measures occurred during active treatment

### Prostate cancer outcomes

#### Binary testosterone exposure: Treated vs. not treated

A total of 313 aggressive prostate cancer events were diagnosed. Among untreated men, there were 190 events (incidence rate (IR) 0.57 per 1000 person years, 95% confidence interval (CI) 0.49–0.65); among testosterone-treated men, there were 123 events (IR 0.58 per 1000 person years, 95% CI 0.48–0.69) (Table 3). With baseline adjustment for age, race, care site, BMI, and recent hospitalization, and time-varying adjustment for medical comorbidities, repeated testosterone testing and PSA screening intensity, there was no difference in risk of aggressive prostate cancer in testosterone-treated versus untreated men: hazard ratio (HR) 0.89; 95% CI 0.70–1.13 (Table 4).

**Table 3:** Person-years of follow-up and incidence of prostate cancer, per 1000 person years.

		Aggressive Prostate Cancer		Any Prostate Cancer	
	Person Years	Cancer Events	Incidence Rate (95% CI)	Cancer Events	Incidence Rate (95% CI)
**Binary T treatment**					
Not treated	335,878	190	0.57 (0.49–0.65)	848	2.52 (2.36–2.70)
Treated	212,719	123	0.58 (0.48–0.69)	591	2.78 (2.56–3.01)
**Cumulative Dose (mg)**					
1–399	37,603	28	0.74 (0.51–1.08)	103	2.74 (2.26–3.32)
400–799	33,841	20	0.59 (0.38–0.92)	82	2.42 (1.95–3.01)
800–1599	39,521	28	0.71 (0.49–1.03)	113	2.86 (2.38–3.44)
1600–3199	38,599	26	0.67 (0.46–0.99)	125	3.24 (2.71–3.86)
≥ 3200	45,750	15	0.33 (0.20–0.54)	124	2.71 (2.27–3.23)

**Table 4:** Association between testosterone treatment and prostate cancer.

	**Aggressive Prostate Cancer**		**Any Prostate Cancer**	
Binary T Treatment*				
No. subjects	147,593		147,593	
No. events	313		1,439	
	**Unadjusted HR (95% CI)**	**Adjusted HR (95% CI)**	**Unadjusted HR (95% CI)**	**Adjusted HR (95% CI)**
Not treated	1.0 (ref)	1.0 (ref)	1.0 (ref)	1.0 (ref)
Treated	1.01 (0.80–1.27)	0.89 (0.70–1.13)	1.10 (0.99–1.22)	0.90 (0.81–1.01)
**Cumulative Dose (mg)**^#^				
No. subjects	56,833		56,833	
No. events	117		547	
	**Unadjusted HR (95% CI)**	**Adjusted HR (95% CI)**	**Unadjusted HR (95% CI)**	**Adjusted HR (95% CI)**
1–399	1.0 (ref)	1.0 (ref)	1.0 (ref)	1.0 (ref)
400–799	0.80 (0.45–1.41)	0.78 (0.44–1.38)	0.88 (0.66–1.18)	0.84 (0.63–1.12)
800–1599	0.96 (0.57–1.62)	0.86 (0.50–1.45)	1.04 (0.79–1.36)	0.90 (0.69–1.18)
1600–3199	0.91 (0.53–1.56)	0.78 (0.46–1.34)	1.18 (0.91–1.53)	0.94 (0.72–1.23)
≥ 3200	0.43 (0.23–0.80)	0.34 (0.18–0.64)	1.02 (0.78–1.32)	0.72 (0.55–0.95)

*Adjusted for baseline age, race, BMI, geography, hospitalization, specific medical morbidities, number of medical morbidities, and time-varying changes in medical morbidities, testosterone testing, PSA screening

^**#**^ Adjusted for baseline age, race, BMI, geography, hospitalization, specific medical morbidities, number of medical morbidities, and time-varying changes in medical morbidities, PSA screening and time from cohort entry to testosterone treatment

A total of 1,439 any prostate cancers were diagnosed; 848 were diagnosed among untreated (IR 2.52 per 1000 person years, 95% CI 2.36–2.70) and 591 among testosterone-treated men (IR 2.78, 95% CI 2.56–3.01). In adjusted analyses, there was no difference in risk of any prostate cancer in testosterone-treated versus untreated men: HR 0.90; 95% CI 0.81–1.01.

#### Cumulative dose among testosterone-treated

In fully-adjusted analyses, there was no association between increasing cumulative testosterone dose and increasing risk of aggressive prostate cancer in testosterone-treated men. However, the highest (≥ 3200 mg) cumulative dose category was associated with a lower risk for aggressive prostate cancer (HR 0.34, 95% CI 0.18–0.64) and for any prostate cancer (HR 0.72, 95% CI 0.55–0.95) compared with the lowest dose category (1–399 mg). (Table 4).

#### Type of testosterone: Intramuscular and topical formulations

Among IM-treated men, there was no increased risk for aggressive (adjusted HR 0.93; 95% CI 0.68–1.27) or any prostate cancer (adjusted HR 0.99; 95% CI 0.86–1.14), relative to untreated men (Table 5). Cumulative dose of IM treatment was not associated with risk of aggressive prostate cancer (S1 Table). It was also not associated with risk of any prostate cancer except the highest cumulative dose (HR 0.61, 95% CI 0.40–0.94) was associated with lower risk of any prostate cancer relative to the lowest dose (S2 Table).

**Table 5:** Association between testosterone treatment and prostate cancer among individuals using single type of formulation.

	**Aggressive Prostate Cancer**		**Any Prostate Cancer**	
**T treatment**	**Intramuscular only**	**Topical only**	**Intramuscular only**	**Topical only**
No. subjects	116,902	116,901	116,902	116,901
No. events	248	240	1,125	1,062
	**Adjusted HRs (95% CI)**		**Adjusted HRs (95% CI)**	
Not Treated	1.0 (ref)	1.0 (ref)	1.0 (ref)	1.0 (ref)
Treated	0.93 (0.68–1.27)	0.92 (0.67–1.26)	0.99 (0.86–1.14)	0.85 (0.73–1.00)

Adjusted for baseline age, race, BMI, geography, hospitalization, specific medical morbidities, number of medical morbidities, and time-varying changes in medical morbidities, testosterone testing, PSA screening

Among topically-treated men, there was no increased risk for aggressive (adjusted HR 0.92; 95% CI 0.67–1.26) or any prostate cancer (adjusted HR 0.85; 95% CI 0.73–1.00) relative to untreated men (Table 5). Cumulative dose of topical testosterone treatment was not associated with risk of aggressive or any prostate cancer (S1 and S2 Tables).

Sensitivity analyses, considering separately men who at cohort entry were aged under 70 years and men who were aged 70 years or older, yielded results consistent with those of the primary analyses. Sensitivity analyses that extended the survival requirement, commencing follow-up at 3 years and 5 years (58,763 men, with 20,776 treated, median follow-up 7.5 years) after cohort entry, also yielded results consistent with those of the primary analyses (Table 6).

**Table 6:** Association between testosterone treatment and any prostate cancer with additional survival requirements.

	**Overall analysis**	**Survival ≥3 years after cohort entry**	**Survival ≥5 years after cohort entry**
Binary T Treatment*	Subjects: 147,953 Events: 1,439	Subjects: 93,609 Events: 831	Subjects: 58,763 Events: 423
	**Adjusted HR (95% CI)**	**Adjusted HR (95% CI)**	**Adjusted HR (95% CI)**
Not treated	1.0 (ref)	1.0 (ref)	1.0 (ref)
Treated	0.90 (0.81–1.01)	0.84 (0.73–0.97)	0.85 (0.69–1.03)
**Cumulative Dose (mg)**^#^	Subjects: 56,833 Events: 547	Subjects: 33,875 Events: 285	Subjects: 20,776 Events: 138
	**Adjusted HR (95% CI)**	**Adjusted HR (95% CI)**	**Adjusted HR (95% CI)**
1–399	1.0 (ref)	1.0 (ref)	1.0 (ref)
400–799	0.84 (0.63–1.12)	0.87 (0.59–1.33)	1.31 (0.74–2.34)
800–1599	0.90 (0.69–1.18)	0.88 (0.59–1.30)	0.87 (0.47–1.62)
1600–3199	0.94 (0.72–1.23)	0.90 (0.62–1.32)	1.23 (0.70–2.17)
≥ 3200	0.72 (0.55–0.95)	0.67 (0.48–0.96)	0.70 (0.42–1.19)

*Adjusted for baseline age, race, BMI, geography, hospitalization, specific medical morbidities, number of medical morbidities, and time-varying changes in medical morbidities, testosterone testing, PSA screening

^**#**^ Adjusted for baseline age, race, BMI, geography, hospitalization, specific medical morbidities, number of medical morbidities, and time-varying changes in medical morbidities, PSA screening and time from cohort entry to testosterone treatment

## Discussion

In analyses adjusted for time-varying medical comorbidities, repeat testosterone testing, and intensity of PSA monitoring, and that followed men for a median of 3.0 years, we observed that in men with low testosterone levels and normal PSA levels at baseline, testosterone treatment was not associated with increased incidence of aggressive or any prostate cancer compared with untreated men. Among men who initiated treatment, we found no evidence of increased aggressive or any prostate cancer risk with increasing cumulative dose of testosterone treatment, and when our analysis was limited to men who were treated only with intramuscular or topical (transdermal) testosterone.

These findings are important given the rise in testosterone treatment and concerns for its potential associated risks. Given the association between testosterone treatment and increase in PSA levels and prostate biopsy, information on the near term risk of prostate cancer among this group of men is important for clinical decision making. In hypogonadal men, testosterone treatment increases prostate size and PSA.[15, 16] Exogenous testosterone has been shown to stimulate growth of metastatic prostate cancer,[24] and androgen-deprivation is used to treat metastatic and locally advanced prostate cancer.[18] However, it is unclear if testosterone treatment increases the risk for incident prostate cancer or if it alters the natural history of pre-existing organ-confined prostate cancer. Recent meta-analyses of placebo-controlled testosterone treatment studies[19, 23, 25] found conflicting results on the association of testosterone treatment with prostate cancer risk.

Observational studies[10, 11, 26] found no increase in aggressive or any prostate cancer in testosterone-treated men but their conclusions were limited by the absence of an appropriate comparison group, lack of adjustment for PSA screening and lack of cumulative dosage analyses. A Swedish nested case-control study[9] found that testosterone treatment was associated with an increase in low and intermediate-risk prostate cancer during the first year of treatment and a decreased risk of aggressive prostate cancer. The former may be due to pre-existing prostate cancer that was detected due to PSA monitoring during initial testosterone treatment, a group that we intentially excluded form our analysis for this specific reason. While this study was the first to suggest that testosterone treatment was associated with a decreased risk for aggressive prostate cancer interpretation is challenging given the lack of data on testosterone levels and frequency of PSA assessment in both cases and controls. Nonetheless, results from our study are consistent with these findings in that among testosterone-treated men, those in the highest dose category (i.e. longest duration of therapy) demonstrated decreased risk for both aggressive and any prostate cancer compared with those in the lowest dose category. However, given few aggressive prostate cancers in the highest cumulative dose group and no consistent association of increasing testosterone dose with decreased risk for aggressive or any prostate cancer, we are reluctant to conclude that higher cumulative doses are associated with decreased aggressive prostate cancer risk.

Our study cohort was selected to be similar to that of a clinical trial of testosterone treatment: no testosterone treatment in the prior year; no previous diagnosis of prostate or breast cancer; and a serum PSA level < 4 ng/dL. To control for potential differences between testosterone-treated and untreated men, we performed time-varying adjustments for 26 medical comorbidities, repeat testosterone testing, and the intensity of PSA screening as these factors may have affected the likelihood of being treated with testosterone and detecting prostate cancer.

We analyzed only men who survived one year after entering the cohort (binary testosterone exposure analysis) or one year after initiating testosterone treatment (cumulative dose analysis) because we assumed that testosterone treatment may lead to increased screening and detection of occult prostate cancer in the first year of treatment. We chose aggressive prostate cancer as the primary outcome because it is the most clinically impactful, leading to higher morbidity and mortality than non-aggressive prostate cancer.[27]

### Strengths and limitations

Our study is the largest pharmaco-epidemiologic study to examine the association between testosterone treatment and prostate cancer. Men were selected for having low testosterone levels and normal PSA levels, a population that is often treated with testosterone, although clinical manifestations of androgen deficiency were not available. A large number of prostate cancers were observed. Data were coded at clinical encounters, not through abstraction. Baseline testosterone and PSA were documented on all men and most treated men had follow-up testosterone and PSA levels. Many subjects received IM testosterone, which has more predictable bioavailability than topical testosterone. All prostate cancers were histologically-confirmed with tumor grade and stage assessment. Furthermore, we adjusted for repeat testosterone testing, PSA screening and changing medical comorbidity over time.

There are limitations of the study. Despite extensive adjustments, residual confounding is possible. We approached confounding adjustment in a time-dependent manner using 26 medical conditions. Alternative analytic methods, such as propensity scores that address the imbalance of characteristics in those who did and did not receive treatment, are possible, but with a large number of subjects and events, propensity scores do not to offer advantages over time-dependent multivariable adjustment [28]. It is feasible that some men have been treated with testosterone outside of the VA, especially those men who are Medicare eligible. Given our inability to assess for non-VA prescribed testosterone use, these individuals may be miscategorized, therefore attenuating our results toward the null hypothesis. Because of the slow progression of prostate cancer, with autopsy studies suggesting that it may take 7–10 years for a prostate cancer to progress from indolent to aggressive, the isolation of men with longer follow-up time is desirable when assessing the risk of testosterone. In our study, median follow-up is 3 years, with the longest follow up time in our cohort being 9.8 years. However, sensitivity analyses that restricted the analytic cohort survival time to a *minimum* of five years were consistent with our primary analyses.

## Conclusions

Among men with low testosterone and normal PSA levels who were followed for a median of 3 years, compared with untreated men, men treated with testosterone were not at increased risk of incident aggressive or any prostate cancer. The longer term clinical risks and benefits of testosterone treatment can only be definitively addressed by large, long-term randomized controlled trials.

## Appendix A. Inclusion and exclusion criteria and censoring criteria

1. Age
  Defined as:
    - Age at time of cohort entry
    - 40 to 89 years at time of first low T test
2. Censoring criteria
  Defined as:
    - Censored from follow-up at the earliest of the following:
      - September 30, 2012 (end of study)
      - 730 days after the last in-person VA clinic visit
      - Date of death
      - For the primary outcome of aggressive prostate cancer: diagnosis of non-aggressive prostate cancer (any prostate cancer not meeting criteria of aggressive prostate cancer, as defined in Appendix B)
3. Exclude prevalent or past breast cancer
  Based on:
    - ICD-9 (*International Classification of Diseases* ninth edition*)* codes for breast cancer in the year prior to cohort entry
  Codes used:
    - ICD-9 codes: 174.xx, 175.xx
4. Exclude prevalent or past prostate cancer
  Based on:
    - ICD-9 codes for prostate cancer in the year prior to cohort entry
    - CPT (*Current Procedural Terminology*) codes for prostate cancer treatment in the year prior to cohort entry
    - Histologically-confirmed diagnosis of prostate cancer in the VACCR (*VA Central Cancer Registry*) in the year prior to cohort entry
  Codes used:
    - ICD-9 codes: V10.46, 60.4, 60.5, 60.52, 60.53, 60.62, 60.69, 185.xx, 233.4
    - CPT codes: 55810, 55812, 55815, 55840, 55842, 55845, 55860, 55862, 55865, 55866, 55873, 55876, 3269F, 3270F, 3271F, 3272F, 3273F, 3274F, 4163F, 4164F, 4201F, C1703, C1706, C1708, C1710, G0256, G0261, G9077, G9078, G9079, G9080, G9081, G9082, G9083, G9132, G9133
5. Exclude men with a history of prostate biopsy
  Based on:
    - CPT codes for prostate biopsy
  Codes used:
    - CPT codes: 55700, 55705, 55706, 0137T, G0417, G0418, G0419
6. PSA value > 4.0 ng/dL
  Based on:
    - LOINC (*Logical Observation Identifiers Names and Codes*) laboratory codes
    - Used for exclusions only, not updated after baseline
  Codes used:
    - LOINC codes: 19195–7, 19197–3, 2857–1, 35741–8, 10886–0, 19201–3, 19203–9, 12841–3, 14120–0, 33667–7, 15323–9, 15324–7, 15325–4
7. Testosterone level flagged as low
  Based on:
    - LOINC laboratory codes
    - Used for inclusion
  Codes used:
    - LOINC codes: 14913–8, 1639–4, 21555–8, 2986–8, 49041–7, 49042–7, 55519–3, 58835–0, 70239–9, 51005–7, 58716–2, 49042–5, 49043–3, 70240–7, 58952–3, 2990–0, 30123–4, 14914–6, 25987–9, 2991–8, 35225–2, 24125–7, 15432–8, 16286–7, 17686–7

## Appendix B. Covariates

1. Age
  Defined as:
    - Age at time of cohort entry
    - Divided into 5-year age categories (years)
      - 40–44; 45–49; 50–54; 55–59; 60–64, 65–69; 70–74; 75–79; 80–84; 85+
    - Not updated after baseline
2. Benign Prostatic Hyperplasia (BPH)
  Based on:
    - ICD-9 codes
    - IENs (*VA National Drug Internal Entry Numbers*) for alpha-blockers and 5 alpha-reductase inhibitors for BPH in the year prior to cohort entry
  Codes used:
    - ICD-9 codes: 222.2x, 599.6x, 600.xx, 601.xx, 602.xx, 788.38, 788.41, 788.42, 788.43, 788.61–788.65, 788.69, 788.2x
    - IENs: 15938, 16325, 19649, 8293, 8294, 12741, 16660, 16661, 19648, 8291, 8295, 8838, 8840, 13685, 20071, 8290, 8292, 8837, 8839, 13610, 13611, 15721, 20070, 8296, 8297, 13634, 16128, 9712, 16129, 9711
3. BMI (kg/m^2^)
  Defined as:
    - 5 categories
      - < 18.5
      - 18.5–24.9
      - 25.0–29.9
      - 30.0–34.9
      - ≥ 35
  Based on:
    - BMI recorded closest to cohort entry date, with preference for prior to cohort entry
    - Updated after baseline only with ICD-9 codes for obesity
  Codes used:
    - ICD-9 codes: V85.4x, 278.01
4. Cardiac Arrhythmia
  Based on:
    - ICD-9 codes
  Codes used:
    - ICD-9 codes: 426.xx, 427.xx
5. Cardiomyopathy
  Based on:
    - ICD-9 codes
  Codes used:
    - ICD-9 codes: 425.xx
6. Cerebrovascular Disease (includes hard/soft outcomes)
  Based on:
    - ICD-9 codes
  Codes used:
    - ICD-9 codes: 38.11, 430.x, 431.x, 433.x1, 434.x1
7. Chronic Heart Failure
  Based on:
    - ICD-9 codes
  Codes used:
    - ICD-9 codes: 428.xx, 398.91
8. Chronic Liver Failure
  Based on:
    - ICD-9 codes
  Codes used:
    - ICD-9 codes: 456.0, 456.1, 456.2x, 571.2, 571.3, 571.40, 571.41, 571.42, 571.49, 571.5, 571.6, 571.8, 571.9, 572.2, 572.3, 572.4, 572.8
9. Chronic Lung Disease
  Based on:
    - ICD-9 codes
  Codes used:
    - ICD-9 codes: 491.2x, 492.xx, 493.xx (NOT 493.81 or 493.82), 496.xx
10. Chronic Pain
  Based on:
    - ICD-9 codes
    - IENs for long-acting opiates (methadone, long-acting morphine, fentanyl) in the 90 days prior to cohort entry
  Codes used:
    - ICD-9 codes: 338.2x
    - IENs: 142, 154, 15712, 15714, 160, 162, 17264, 20101, 378, 5800, 150, 15361, 156, 159, 21479, 387, 5799, 136, 147, 151, 152, 153, 15362, 155, 15703, 161, 163, 164, 165, 16557, 17697, 394, 396, 5801, 138, 139, 141, 143, 148, 149, 157, 15763, 158, 16556, 377, 5802
11. Coronary Artery Disease (includes hard/soft outcomes)
  Based on:
    - ICD-9 codes
    - CPT codes
  Codes used:
    - ICD-9 codes: 36.0x, 36.1x, 36.2x, V45.81, V45.82, 410.xx, 411.1, 411.8, 411.81, 411.89, 412.xx, 413.x, 414.xx (NOT 414.1x)
    - CPT codes: 33510, 33511, 33512, 33513, 33514, 33516, 33517, 33518, 33519, 33521, 33522, 33523, 92980, 92981, 92982, 92984, 92985
12. Diabetes
  Based on:
    - ICD-9 codes
    - IENs for oral hypoglycemics and insulin in the year prior to cohort entry
    - LOINC laboratory codes for HbA1c > 6 in the year prior to cohort entry
  Codes used:
    - ICD-9 codes: 250.xx, 362.0x
    - IENs: 22955, 17238, 17239, 14371, 14913, 16264, 16280, 17587, 18029, 19354, 20717, 822, 826, 829, 831, 834, 838, 841, 845, 846, 850, 852, 858, 862, 863, 868, 873, 874, 879, 880, 886, 888, 889, 891, 13113, 13485, 16265, 16466, 16467, 16746, 17588, 18242, 18243, 18690, 823, 824, 828, 836, 837, 842, 844, 853, 856, 867, 869, 871, 875, 882, 885, 890, 13351, 13484, 14591, 14592, 16199, 16200, 16437, 16665, 17851, 18590, 19355, 825, 827, 839, 840, 851, 854, 855, 857, 859, 860, 864, 866, 870, 877, 881, 883, 884, 887, 13352, 13353, 13486, 14476, 14477, 16263, 16370, 17849, 17850, 17894, 19356, 20714, 821, 830, 832, 833, 835, 843, 847, 848, 849, 861, 865, 872, 876, 878, 892, 12469, 12485, 13579, 14537, 15347, 15983, 16137, 17220, 1780, 18086, 18282, 1836, 19238, 2079, 21861, 22043, 22947, 23321, 23629, 23631, 23632, 23638, 23797, 4519, 12369, 12470, 12472, 12483, 12484, 12594, 12766, 13507, 13508, 13580, 13581, 13712, 13713, 13714, 14538, 14620, 15981, 15982, 16125, 16139, 17926, 18281, 1837, 19239, 2078, 20859, 22946, 22948, 22959, 23796, 2877, 576, 12468, 12471, 12592, 12593, 12768, 12901, 12976, 13875, 14539, 14941, 16635, 16711, 1779, 1781, 1782, 17912, 18006, 18009, 20177, 21862, 22042, 22389, 22732, 22733, 22958, 22960, 23333, 23623, 23624, 23630, 23637, 2876, 2879, 2880, 4518, 4520, 4521, 12370, 12767, 12899, 12900, 13509, 14319, 14940, 16124, 16126, 16138, 17191, 17542, 17543, 17925, 17927, 18007, 18008, 18010, 18011, 18085, 19240, 20559, 20561, 20858, 22041, 22731, 23332, 23622, 23628, 23633, 2878, 2881, 577, 21531, 17188, 19123, 19375, 19124, 19125
    - LOINCs: 41995–2, 55454–3, 4548–4, 4549–2, 17855–8, 17856–6, 59261–8, 71875–9, 62388–4
13. Frailty
  Based on:
    - ICD-9 codes
    - BMI recorded closest to cohort entry is < 18.5
  Codes used:
    - ICD-9 codes: 728.2x, 783.7x, 797.xx
14. Geographic region
  Defined as:
    - The “Home VISN” (*Veterans Integrated Service Network*) listed in the patient’s record closest to cohort entry date
    - Not updated after baseline
15. Hospitalization in past year
  Defined as:
    - Inpatient hospitalization within the 365 days prior to the cohort entry date
16. Hyperlipidemia
  Based on:
    - ICD-9 codes
    - IENs for lipid-lowering medications in the year prior to cohort entry
  Codes used:
    - ICD-9 codes: 272.0x, 272.1x, 272.2x
    - IENs: 13591, 18377, 22009, 5850, 5851, 5854, 5856, 5859, 5861, 5862, 5864, 6227, 13589, 18376, 18378, 21539, 22008, 22010, 5846, 5853, 5860, 6223, 13592, 17507, 21541, 5847, 5855, 5857, 5858, 6226, 13588, 13590, 21540, 5848, 5849, 5852, 5863, 6224, 6225, 11937, 12860, 12861, 13578, 14096, 14585, 14760, 15318, 15470, 15866, 16386, 16389, 16400, 16403, 16531, 16775, 16776, 16880, 17012, 17338, 17552, 18969, 19385, 21013, 23564, 2907, 5098, 5238, 6115, 6116, 6118, 7994, 12596, 14759, 15317, 15865, 16004, 16390, 16392, 16405, 16442, 16532, 16877, 16879, 16882, 16883, 16885, 17013, 17335, 17336, 19374, 19386, 19387, 19510, 19636, 20339, 21312, 21729, 23572, 23859, 23860, 2906, 2908, 2909, 6114, 6119, 8519, 9680, 9741, 12595, 12597, 13788, 14321, 14618, 16135, 16244, 16387, 16391, 16404, 16881, 16884, 18040, 19527, 20340, 21014, 21310, 21311, 21867, 23735, 23858, 2910, 5239, 6117, 8520, 9743, 9744, 11938, 12831, 12914, 13725, 14527, 14594, 14960, 15864, 16003, 16388, 16401, 16402, 16530, 16777, 16878, 17334, 17337, 18970, 19366, 19526, 19637, 21509, 21866, 23563, 23565, 23864, 4436, 5097, 6113, 8521, 9681, 9682, 9742
17. Hypertension
  Based on:
    - ICD-9 codes
  Codes used:
    - ICD-9 codes: 401.xx, 402.xx, 403.xx, 404.xx, 405.xx
18. Major Depression
  Based on:
    - ICD-9 codes
  Codes used:
    - ICD-9 codes: 296.2x-296.3x
19. Malignancy
  Based on:
    - ICD-9 codes
  Codes used:
    - ICD-9 codes: 140.xx-239.xx (NOT 140.xx, 173.xx, 174.xx, 179.xx, 180.xx-184.xx and 210.xx-234.xx)
20. Morbid Obesity
  Based on:
    - ICD-9 codes
  Codes used:
    - ICD-9 codes: V85.4x, 278.01
21. Osteoporosis
  Based on:
    - ICD-9 codes
  Codes used:
    - ICD-9 codes: 733.00, 733.01, 733.02, 733.09
22. Peripheral Vascular Disease
  Based on:
    - ICD-9 codes
    - CPT codes
  Codes used:
    - ICD-9 codes: 38.18, 38.19, 38.38, 38.39, 38.48, 38.49, 38.88, 38.89, 39.25, 39.26, 39.28, 39.29, 39.50, 39.90, 433, 433.9, 440.2x, 440.3x, 440.4x, 442.x, 443.x, 445.0x
    - CPT codes: 34800, 34802, 34803, 34804, 34805, 35226, 35256, 35286, 35351, 35355, 35371, 35372, 35381, 35454, 35456, 35459, 35473, 35474, 35482, 35483, 35485, 35492, 35493, 35495, 35546, 35548, 35549, 35551, 35556, 35558, 35563, 35565, 35566, 35571, 35583, 35585, 35587, 35646, 35656, 35661, 35663, 35665, 35666, 35671
23. Polycythemia
  Based on:
    - ICD-9 codes
    - LOINC laboratory codes for HCT > 52 in the year prior to cohort entry
  Codes used:
    - ICD-9 codes: 289.0x
    - LOINCs: 24360–0, 4544–3, 71833–8, 4545–0, 48703–3, 20570–8, 41655–2, 71830–4, 31100–1
24. PSA (Prostate Specific Antigen) screening
  Defined as:
    - 3 categories
      - Not screened in the last 12 months
      - Screened in the last 6–12 months
      - Screened in the last 6 months
  Based on:
    - Date of most recent PSA measure
    - LOINC laboratory codes
  Codes used:
    - LOINC codes: 19195–7, 19197–3, 2857–1, 35741–8, 10886–0, 19201–3, 19203–9, 12841–3, 14120–0, 33667–7, 15323–9, 15324–7, 15325–4
25. Race
  Defined as:
    - 3 categories
      - Black
      - White
      - Other
    - Not updated after baseline
26. Repeat T levels
  Defined as:
    - 3 categories
      - No repeat T measure
      - First repeat T level low
      - First repeat T level non-low
    - Repeat T levels prior to treatment initiation only
    - T level updated only at first repeat T level
  Based on:
    - LOINC laboratory codes
  Codes used:
    - LOINC codes: 14913–8, 1639–4, 21555–8, 2986–8, 49041–7, 49042–7, 55519–3, 58835–0, 70239–9, 51005–7, 58716–2, 49042–5, 49043–3, 70240–7, 58952–3, 2990–0, 30123–4, 14914–6, 25987–9, 2991–8, 35225–2, 24125–7, 15432–8, 16286–7, 17686–7
27. Sexual Dysfunction
  Based on:
    - ICD-9 codes
    - IENs for PDE5 inhibitors for ED and other ED drugs in the year prior to cohort entry
  Codes used:
    - ICD-9 codes: 302.70, 302.71, 302.72, 302.74, 302.75, 302.76, 607.84, 799.81
    - IENs: 16380, 16384, 12823, 16381, 16385, 16522, 20312, 22644, 12822, 12824, 16520, 16521, 16379, 16382, 16383, 16146, 2956, 2957, 2962, 2963, 2954, 2961, 2964, 16147, 16509, 2955, 2965, 16148
28. Sleep Apnea
  Based on:
    - ICD-9 codes
  Codes used:
    - ICD-9 codes: 780.51, 780.53, 780.57
29. Smoking
  Based on:
    - ICD-9 codes
    - IENs for smoking cessation drugs in the year prior to cohort entry
  Codes used:
    - ICD-9 codes: V15.82, 305.1x, 989.84
    - IENs: 17847, 23100, 9697, 9703, 16376, 18746, 18749, 22944, 5095, 9700, 9701, 16375, 17845, 18747, 5096, 9696, 13203, 16685, 17846, 17848, 18748, 22943, 9694, 9695, 9698, 9699, 9702
30. Testosterone exposure
  Based on:
    - Cumulative exposure level
    - Accumulates at the end of each prescription
    - 5 categories
      - 1–399 mg
      - 400–799 mg
      - 800–1599 mg
      - 1600–3199 mg
      - 3200+ mg
    - Based on IENs for testosterone
    - Based on pharmacy data
  Codes used:
    - IENs: 513, 514, 515, 516, 518, 524, 525, 526, 527, 530, 531, 532, 533, 534, 14379, 14380, 14775, 15507, 15508, 16064, 16544, 17475, 17503, 17901, 21468, 21470, 21471, 22219, 22384, 22523, 22526, 22791, 168, 169, 170, 171, 172, 173, 174, 512, 517, 519, 520, 521, 522, 523, 528, 529, 1301, 1302, 1303, 3055, 3637, 3638, 3639, 3795, 4220, 4550, 4551, 4552, 4553, 4554, 4555, 4556, 6577, 6578, 6976, 16141
31. Testosterone Formulation
  Based on:
    - IENs
    - Pharmacy data
  Codes used:
    - IENs: 513, 514, 515, 516, 518, 524, 525, 526, 527, 530, 531, 532, 533, 534, 14379, 14380, 14775, 15507, 15508, 16064, 16544, 17475, 17503, 17901, 21468, 21470, 21471, 22219, 22384, 22523, 22526, 22791, 168, 169, 170, 171, 172, 173, 174, 512, 517, 519, 520, 521, 522, 523, 528, 529, 1301, 1302, 1303, 3055, 3637, 3638, 3639, 3795, 4220, 4550, 4551, 4552, 4553, 4554, 4555, 4556, 6577, 6578, 6976, 1614
32. Traumatic Brain Injury (TBI)
  Based on:
    - ICD-9 codes
  Codes used:
    - ICD-9 codes: 310.2x, 801.xx, 800.xx, 802.xx, 803.xx, 804.xx
33. Venous Thrombosis and Pulmonary Embolism
  Based on:
    - ICD-9 codes
    - IENs for blood thinners used for DVT in the year prior to cohort entry
  Codes used:
    - ICD-9 codes: 415.1, 415.11, 415.12, 415.13, 415.19, 453.xx
    - IENs: 11785, 12935, 12938, 13678, 15894, 17111, 18874, 18877, 21208, 21213, 21214, 21215, 22021, 22022, 22575, 22765, 4653, 4654, 4658, 4660, 12362, 12932, 13675, 13676, 15440, 15895, 15896, 17183, 18878, 21207, 21209, 21210, 21212, 22573, 22764, 22766, 4652, 4656, 11784, 11787, 12936, 13624, 13625, 13674, 13677, 13679, 15439, 17110, 17112, 18876, 19109, 21211, 22019, 22020, 4650, 4651, 4657, 4659, 11783, 11786, 12360, 12361, 12933, 12934, 14609, 15480, 22763, 4655, 5592, 5593

## Appendix C. Outcome definitions

1. Aggressive Prostate Cancer
  Defined as:
    - Histologically-confirmed prostate cancer only
    - Diagnosed after cohort entry
    - Must be classified as one or more of the following:
      - SEER Summary Stage = 7
      - AJCC Stage Group = 4 or IV
      - Any metastasis
      - Tumor Grade = 4
      - Gleason Score ≥ 8
      - PSA ≥ 20
2. Any Prostate Cancer
  Defined as:
    - Histologically-confirmed prostate cancer only
    - Diagnosed after cohort entry
    - Includes aggressive prostate cancer
  Based on:
    - Data from VACCR

## Supporting information

S1 Fig Study cohort inclusion and exclusion. (DOCX)

S1 Table Association between cumulative dose and aggressive prostate cancer by formulation. (DOCX)

S2 Table Association between cumulative dose and any prostate cancer by formulation. (DOCX)
