# Supplementary material for: Testosterone treatment and the risk of aggressive prostate cancer in men with low testosterone levels
Source: PLoS One. 2018 Jun 22;13(6):e0199194. doi: 10.1371/journal.pone.0199194 (PMC6014638; doi:10.1371/journal.pone.0199194)
Supplement: S1 Fig — (DOCX) [file pone.0199194.s001.docx]

**S1 Figure**. Study Cohort Inclusion and Exclusion

**301,003** men in the VA system who had a testosterone (T) level measured and flagged as low between January 1, 2002 and December 31, 2011 AND were aged 40-89 at the time of the low T test AND had complete lab data

**153,410** men excluded

1,132 men with insufficient outpatient visits

87,133 men without low T test and Prostate Specific Antigen (PSA) screening within 6 months of one another

40,571 men who initiated T prior to cohort entry

11,412 men with qualifying PSA screening result ≥4 ng/dl

1,642 men with history of prostate cancer, breast cancer, or biopsy or died before cohort entry

1,802 men missing information on covariates used in the analytic models

9,718 men who did not survive one year past cohort entry

**147,593** men were included in treated/not treated analysis

**1,784** men excluded

1,784 men did not survive one year on treatment

15,272 men with maximum cumulative dose > 3200 mg

11,086 men with maximum cumulative dose 1600-3199 mg

10,913 men with maximum cumulative dose 800-1599 mg

9,039 men with maximum cumulative dose 400-799 mg

10,523 men with 0 mg ≤ maximum cumulative dose ≤ 399 mg

**56,833** men were included in cumulative dose analysis

**58,617** men treated with T

**88,976** men not treated with T
