# Supplementary material for: Testosterone treatment and the risk of aggressive prostate cancer in men with low testosterone levels
Source: PLoS One. 2018 Jun 22;13(6):e0199194. doi: 10.1371/journal.pone.0199194 (PMC6014638; doi:10.1371/journal.pone.0199194)
Supplement: S1 Table — (DOCX) [file pone.0199194.s002.docx]

**S1 Table**. Association between cumulative dose and aggressive prostate cancer by formulation

| **T Treatment Cumulative Dose (mg)^#^** | **All** | **Intramuscular only** | **Topical only** |
| --- | --- | --- | --- |
| No. subjects | 56,833 | 25,260 | 24,360 |
| No. events | 117 | 54 | 48 |
|  | **Adjusted HR (95% CI)** | **Adjusted HR (95% CI)** | **Adjusted HR (95% CI)** |
| 1-399 | 1.0 (ref) | 1.0 (ref) | 1.0 (ref) |
| 400-799 | 0.78 (0.44-1.38) | 1.11 (0.44-2.85) | 0.45 (0.18-1.11) |
| 800-1599 | 0.86 (0.50-1.45) | 1.13 (0.47-2.76) | 0.54 (0.23-1.28) |
| 1600-3199 | 0.78 (0.46-1.34) | 0.68 (0.26-1.79) | 0.84 (0.38-1.85) |
| ≥ 3200 | 0.34 (0.18-0.64) | 0.40 (0.14-1.11) | 0.53 (0.19-1.43) |

**#** Adjusted for baseline age, race, BMI, geography, hospitalization, specific medical morbidities, number of medical morbidities, and time-varying changes in medical morbidities, PSA screening and time from cohort entry to testosterone treatment
