# Supplementary material for: Testosterone treatment and the risk of aggressive prostate cancer in men with low testosterone levels
Source: PLoS One. 2018 Jun 22;13(6):e0199194. doi: 10.1371/journal.pone.0199194 (PMC6014638; doi:10.1371/journal.pone.0199194)
Supplement: S2 Table — (DOCX) [file pone.0199194.s003.docx]

**S2 Table**. Association between cumulative dose and any prostate cancer by formulation

| **T Treatment Cumulative Dose (mg)^#^** | **All** | | **Intramuscular only** | | **Topical only** | |
| --- | --- | --- | --- | --- | --- | --- |
| No. subjects | 56,833 | | 25,260 | | 24,360 | |
| No. events | 547 | | 257 | | 197 | |
|  | | **Adjusted HR (95% CI)** | | **Adjusted HR (95% CI)** | | **Adjusted HR (95% CI)** |
| 1-399 | 1.0 (ref) | | 1.0 (ref) | | 1.0 (ref) | |
| 400-799 | 0.84 (0.63-1.12) | | 0.94 (0.60-1.47) | | 0.68 (0.45-1.04) | |
| 800-1599 | 0.90 (0.69-1.18) | | 0.78 (0.50-1.20) | | 0.91 (0.61-1.35) | |
| 1600-3199 | 0.94 (0.72-1.23) | | 0.82 (0.53-1.26) | | 0.95 (0.63-1.44) | |
| ≥ 3200 | 0.72 (0.55-0.95) | | 0.61 (0.40-0.94) | | 0.79 (0.49-1.29) | |

**#** Adjusted for baseline age, race, BMI, geography, hospitalization, specific medical morbidities, number of medical morbidities, and time-varying changes in medical morbidities, PSA screening and time from cohort entry to testosterone treatment
